# Autopsy or anatomical dissection: evidence of a craniotomy in a 17th–eighteenth century burial site (Ravenna, Italy)

**DOI:** 10.1007/s12024-020-00285-6

**Published:** 2020-08-08

**Authors:** Filippo Scianò, Nicoletta Zedda, Jessica Mongillo, Emanuela Gualdi-Russo, Barbara Bramanti

**Affiliations:** Faculty of Medicine, Pharmacy and Prevention, Department of Biomedical Sciences and Surgical Specialties, University of Ferrara, Corso Ercole I, d’Este 32, 44121 Ferrara, Italy

**Keywords:** Craniotomy, Autopsy, Forensic anthropology, Pathology

## Abstract

Surgical procedures undergone in life, autopsies and anatomical preparations can all leave clearly identifiable traces on human skeletal remains. Several studies on skeletons from archeological contexts have identified traces of these practices. However, the distinction between medical/forensic autopsy and anatomical dissections for scientific research can be challenging. We report the case of a middle-aged female skeleton from the cemetery of the church of San Biagio (Ravenna, Italy), dating back to the 17th–19th centuries, that shows signs of a complete craniotomy. In an attempt to clarify the reason for this practice, we analyzed all pathological and non-pathological markers on the skeleton. We carried out anthropological analyses and osteometric measurements to determine the biological profile and the cranial capacity of the individual. Paleopathological investigation and analyses of traumatic injury patterns were carried out using both a morphological and a microscopic approach. While we observed that the craniotomy was performed with a rip saw, we identified perimortem blunt force trauma to the frontal bone and an osteolytic lesion on the inner surface of the frontal bone. No other pathology was recognizable on the skeleton. Our differential diagnosis confidently proved that the craniotomy was due to an autoptsy procedure and was not the result of an anatomical dissection. We believe that, among other possible reasons, failed surgery could likely be the motive behind the ordering of the autopsy.

**Electronic supplementary material:**

The online version of this article (10.1007/s12024-020-00285-6) contains supplementary material, which is available to authorized users.

## Introduction

Several studies on human skeletons from archeological contexts have reported evidence of surgical practices that occurred in life, or traces of post-mortem autopsy examination and anatomical dissection [1, 2]. The differentiation between medical/forensic autopsy cases and anatomical dissections for scientific research is not always specified.

In this study, we describe and discuss the case of a skeletonized individual (US-217) with signs of an alleged autoptsy examination of the skull. The human remains were recovered in the cemetery of the church of San Biagio (Ravenna, Italy) and date back from the 17th to the early nineteenth century. This skeleton was the only one with evidence of putative autopsy or surgical practice from more than 200 individuals recovered in the cemetery.

We carried out an anthropological analysis, examining each lesion (perimortem and postmortem), to determine the probable causes of death of the individual and interpret the evident signs of craniotomy found on the skull.

## Material and methods

The individual under examination is one of the several human skeletal remains from the cemetery of St. Biagio (Ravenna) and was studied in the Laboratory of Archeo-anthropology and Forensic Anthropology of our Institution. We determined the biological profile of individual US-207 following the traditional anthropological methods for sex [3], age at death [3, 4] and height estimations [5, 6]. To determine cranial proportions and estimate cranial capacity, we took osteometric measurements of the skull (maximum cranial length and breadth, basion-bregma height) using a spreading caliper (GPM, Siber Hegner & Co., Ltd., Zurich, Switzerland) [7].

Paleopathological investigation and analysis of traumatic injury pattern were carried out using both a morphological and a microscopic approach. The description and evaluation of traumas were performed to assess the nature (accidental or intentional) and timing of the injuries (ante-mortem, peri-mortem or post-mortem) [8–10], according to the most applied methods in forensic anthropology.

## Results

The skeleton of US-217 belonged to an adult woman with an age at death of between 35 and 50 years and stature in life of 156.4 ± 4.2 cm.

Osteometric measurements showed that US-217 was brachycranic and hypsicranic, with a cranial capacity of 1100–1400 cm^3^, thus without any evident abnormality.

**Fig. 1:**
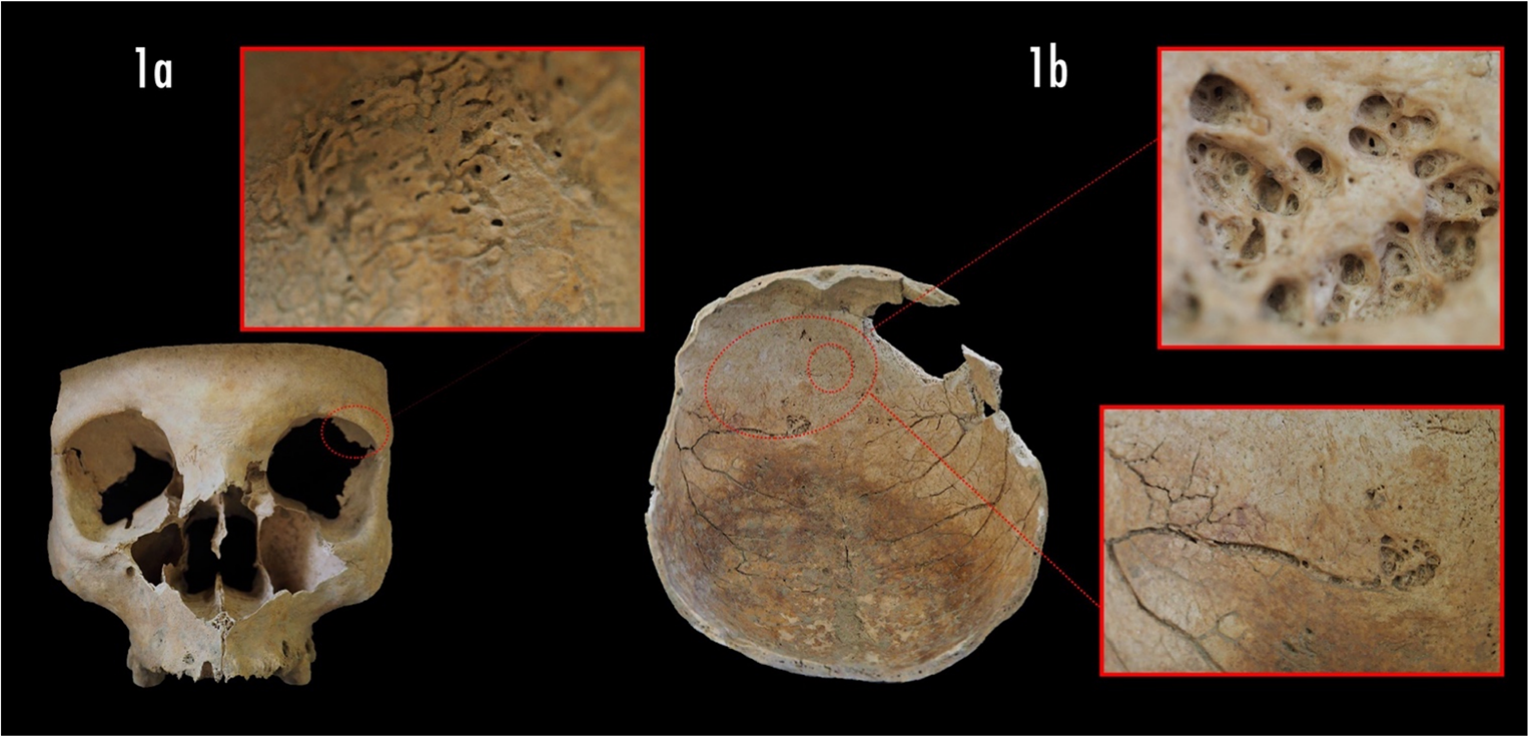
Cranium of the Ind. US 217: **a**) Macro view of the observed cribra orbitalia; **b**) Macro view at different magnifications of the osteolytic lesion on the right frontal bone and of the deep vascular impressions on the endocranial surface linked to the osteolytic lesion

The paleopathological investigation has shown the presence of active cribra orbitalia on the left orbital roof (Fig. 1a) (degree of severity 3; degree of healing 1) [11], that can be indicative of an iron deficiency anemia.

**Fig. 2:**
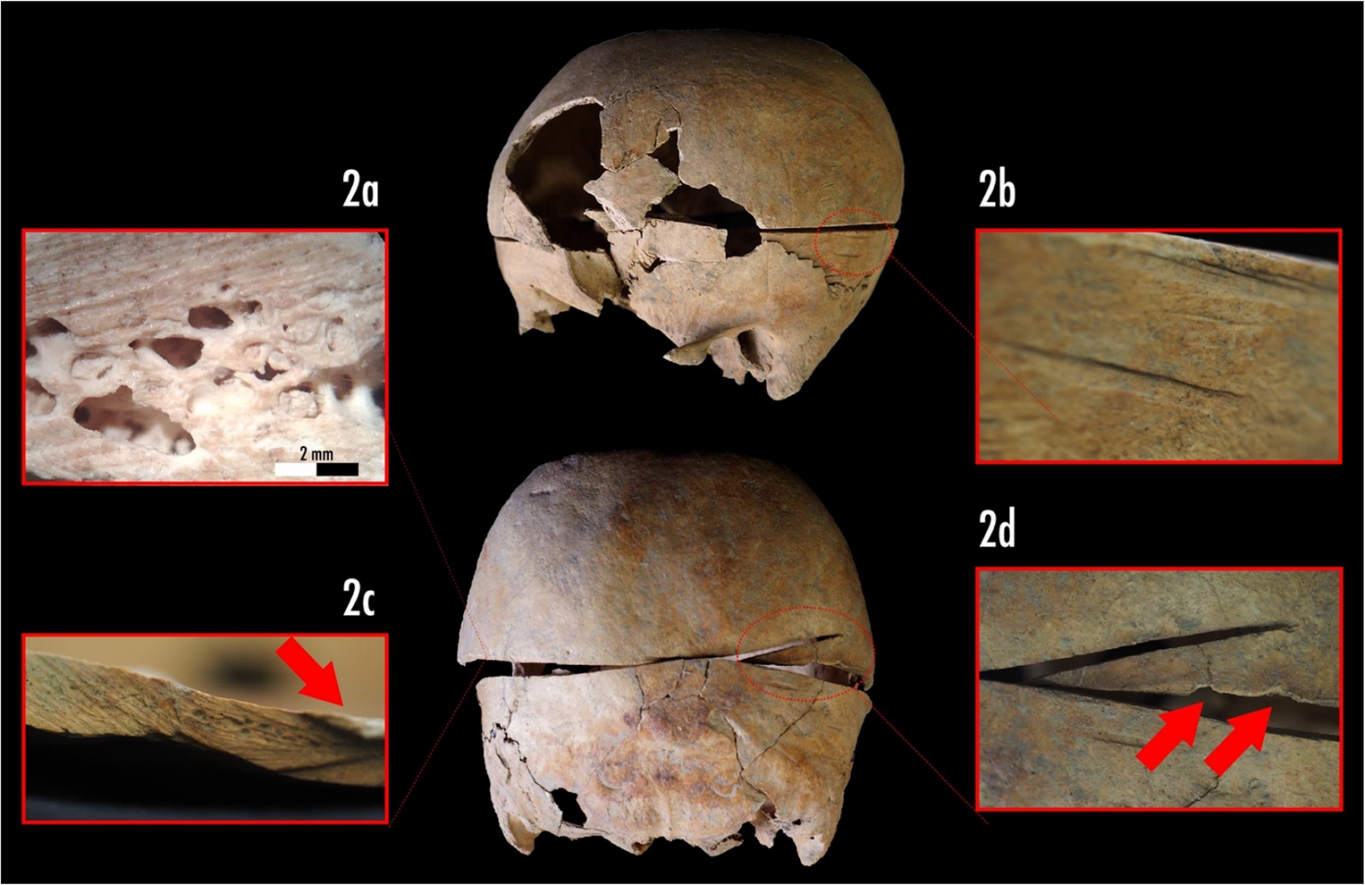
Circumferential craniotomy: **a**) Microscopic view of saw marks pattern (40x magnification); **b**) Macro view, double false-start kerf; **c**) Macro view of saw marks on the kerf floor and convex break away spur (red arrow); **d**) Macro view of touch point between the two side of sawing lines, presence spine, false-start kerf and exit chipping (red arrows)

The most significant traumatic evidence on the skull was due to deep cut marks left by a saw along a transverse plane passing through the *Ophryon - Lambda* landmarks (Fig. 2). On both superior and inferior sides of the sawed skull, the typical markers left by a rip saw were observable. Microscopically, it was possible to recognize all the distinctive traits of a complete craniotomy, among which are: a) the kerf floor with parallel striae left by the teeth of the saw (Fig. 2a); b) the false start kerf (Fig. 2b) that indicates a potential point of departure or resumption of the cutting action; c) the kerf walls with the peculiar break-away spurs (positive) (Fig. 2c, red arrow); and d) the areas of fracture, where the cut ended, with chipping caused by the saw at the exit (Fig. 2d red arrows).

**Fig. 3:**
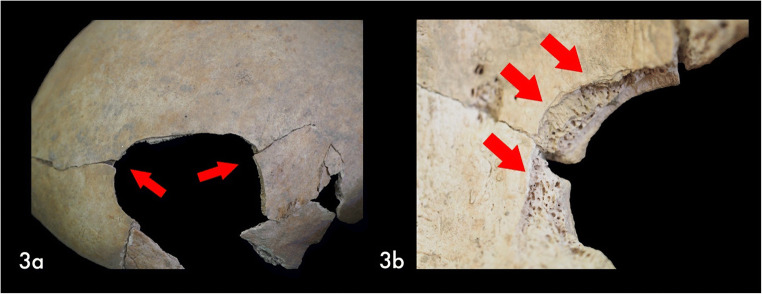
Traumatic evidence on the frontal bone: **a**) Area of circular lesion with probable radial fractures (red arrows); **b**) Macro view of the margin of the lesion – Flakes’ detachment (red arrows) and beveling

Another injury was noticeable on the left side of the frontal bone (Fig. 3). The lesion had an elliptical shape with a maximum diameter of 42.1 mm. The external lamina was not affected by residual traces left by the “weapon”, nor did it show signs of bone reaction (periostitis) or pathologic reaction (osteitis). The margins were sharp and well defined, with no traces of bone remodeling (osteoblastic activity), nor signs of discoloration and other diagenetic changes. Presumed vestiges of radial fractures were visible (Fig. 3a, red arrows). On the endocranial surface, the edges of the lesion were irregular, the diplöe was exposed and the margins were introflexed (Fig. 3b). Moreover, the surface along the lesion was characterized by a typical detachment of small bone flakes (Fig. 3b, red arrows) and internal beveling along the periphery [8].

None of the traumatic lesions on individual US-217’s skull showed any evidence of a healing process. The comparison with the literature [9] and the diagnosis of the timing of the lesions carried out with a specific evaluation form [12] (ESM-Tab. S1) indicated that the traumas occurred at or around the time of death.

Further, on the endocranial surface of the left frontal bone, we detected a singular well identifiable osteolytic lesion of 14 mm in diameter, closely connected to the deep blood vessels (*arteria sulci prefrontali and venae superiores cerebri*) (Fig. 1b).

Neither pathological or traumatic evidence was observed on the post-cranium of this individual.

## Discussion

The large lesion that divided the skull of US-217 into two parts (Fig. 2), given its precision, is due to a scalping procedure of a surgical nature. The coarse striae (angle of cut 90°) on both sides of the sawed bone were most probably produced by a surgical saw with 4.5 teeth per inch [10, 13], along with other complementary tools (e.g. chisels and/or surgical blades).

The presence of specific cut-marks allows the identification of human autopsy practice in archaeological and forensic contexts and could potentially be a good parameter to differentiate it from anatomic dissection. As demonstrated by Dittmar and Mitchell [2], several indicators can be used to distinguish between autopsy practices and anatomical dissections. Using the same indicators [2] for US-217 (Table 1), we concluded that the lesion was the result of a circumferential craniotomy during an autopsy [1, 14] even if no other surgical evidence (e.g. sternotomy or limb disarticulation) has been found on the post-cranium.

**Table 1:** Features compatible with autopsy or dissection in excavated skeletal remains compared with our sample (from Dittmar and Mitchell, 2015 [3]; modified)

	Autopsy	Dissection	Individual US 217
Circumferential craniotomy	✓	✓	✓
Coronal or sagittal craniotomy	✕	✓	✕
Sternotomy or thoracotomy	✓	✓	✕
Knife cuts from defleshing without craniotomy	✕	✓	✕
Sawing of corpse into sections	✕	✓	✕
Non-matching body parts in grave	✕	✓	✕
Wax casts of hollow organs	✕	✓	✕
Coloured dyes on bone	✕	✓	✕
Dissected animal bones in same grave	✕	✓	✕

Legenda: ✕ marker is absent, ✓ marker may be present

Although we could clearly demonstrate that the observed injury was due to an autopsy, the reasons for ordering the autoptic practice still need to be clarified.

The circumscribed lesion on the frontal bone is compatible with a peri-mortem blunt force trauma (BFT). Nonetheless, the absence of any marks left by the tool did not allow us to confirm it and accurately identify the “weapon” used. In fact, this lesion could have been the cause of death of US-217, or it could be interpreted as the result of an attempt of a decompression surgery carried out to treat acute head pain or other symptoms the patient suffered. We have noticed traces of a lytic pathologic lesion on the endocranial surface of the frontal bone (Fig. 1a). We could speculate that the woman suffered from head pain, sudden mood swings or seizures, therefore, she might have undergone surgery to relieve the symptoms. The surgery was unsuccessful and, after her death, the physician might have ordered the autopsy for anatomic-pathological investigation.

## Conclusion

In the 17th–19th centuries, the autopsy was a common practice, albeit frowned upon, for anatomic-pathological investigations. Similar cases of autoptic intervention have been recorded in different archaeological sites of the Italian peninsula [14, 15], showing similarities to those of individual US-217. Yet, the other cases were always found in hospital cemeteries and associated with bone assemblages presenting the same features. The singularity of our discovery allows us to conclude that autopsy was unconventional in Emilia Romagna at that time, and that it might have been practiced in this singular case to better understand the origin of a particular symptomatology which, perhaps, had never been observed before.

## Electronic supplementary material

ESM 1 (PDF 260 kb)
