# Supplementary material for: Autopsy or anatomical dissection: evidence of a craniotomy in a 17th–eighteenth century burial site (Ravenna, Italy)
Source: Forensic Sci Med Pathol. 2020 Aug 8;17(1):157–60. doi: 10.1007/s12024-020-00285-6 (PMC7889548; doi:10.1007/s12024-020-00285-6)
Supplement: Supplementary file 1 — (PDF 260 kb) [file 12024_2020_285_MOESM1_ESM.pdf]

Electronic Supplementary Material

Tab. S1 – Features of the lesions found in Individual US-217 and their timing. The analyses were carried out with the method and the form of Scianò et al. 2020 [47].

Archaeological site: Church of San Biagio (Ravenna)			Individual: US 217
**Type of lesion**	**Time of trauma**	**Healing process**	**Bony responses**
**Circumferential lesion**	Perimortem	Absence of bony response or healing	• Edges of injuries irregular and shaped; hinging bone fragments.<br>• Cortical bone flakes along fracture margins.<br>• Internal or/and external smoothed margins.<br>• Radiating and concentric fractures.<br>• Staining maintained along fracture margins.<br>• Plastic deformation.
**Lesion of frontal bone**	Perimortem	Absence of bony response or healing	• Edges of injuries irregular and shaped; hinging bone fragments.<br>• Cortical bone flakes along fracture margins.<br>• Internal or/and external smoothed margins.<br>• Radiating and concentric fractures.<br>• Staining maintained along fracture margins.
